# Subpial hemorrhage: A rare and underrecognized site of intracranial hemorrhage in neonates

**DOI:** 10.1016/j.radcr.2024.11.088

**Published:** 2024-12-27

**Authors:** Anass El M Aary

**Affiliations:** From the Department of Radiology, University of Bourgogne Franche-Comté, CHRU Besançon, 3 boulevard Alexandre Fleming, Besançon 25030, France

**Keywords:** Subpial hemorrhage, Neonatal hemorrhagic lesions, Neonatal seizures

## Abstract

Subpial hemorrhage (SPH) is a rare but significant cause of neonatal seizures and respiratory distress, primarily affecting full-term infants without apparent risk factors. We report the case of a full-term newborn who presented with recurrent episodes of apnea, desaturation, and seizures shortly after birth. MRI revealed an acute hemorrhagic collection in the left temporal region, accompanied by cortical cytotoxic edema. The diagnosis of SPH was established based on characteristic imaging findings. The patient was treated with anticonvulsant therapy and intensive neonatal care, leading to a favorable outcome. Early diagnosis through neuroimaging, particularly MRI, is crucial for proper management, as SPH may be missed on routine ultrasound. Despite its rarity, SPH generally carries a good prognosis with timely intervention, although larger hemorrhages or cases in preterm infants may have a more uncertain prognosis.

## Introduction

Subpial hemorrhage (SPH) is a rare form of intracranial hemorrhage that occurs in the subpial space—a region situated between the subarachnoid space and the cortical surface. Despite its rarity, SPH is important to recognize as it can occur in neonates presenting with seizures, respiratory distress, or unexplained neurological symptoms. Its diagnosis relies on neuroimaging, particularly MRI.

SPH typically affects full-term infants without apparent risk factors, although it has been associated with acute perinatal events such as fetal distress. The condition is characterized by subpial hematomas accompanied by cortical cytotoxic edema and ischemic changes in the underlying white matter. Prompt recognition and management are critical, as the prognosis is generally favorable with appropriate neonatal intensive care and anticonvulsant therapy. This case report highlights a neonate with SPH, emphasizing the clinical and radiological findings, management approach, and outcomes, while shedding light on this underrecognized condition.

## Case report

We present the case of a full-term male infant born at 38 weeks and 6 days of gestation with a birth weight of 3200g (54th percentile). The newborn's Apgar scores were 10-10-10 at 1, 5, and 10 minutes, indicating good adaptation to extrauterine life. However, shortly after birth, the infant developed hypothermia with a body temperature of 35.6°C, necessitating hospitalization in the maternity ward.

Few hours latter, a midwife reported that the infant appeared cyanotic during a routine temperature check. Initial monitoring in the maternity ward revealed normal oxygen saturation levels of 97%. However, the infant soon experienced a deep desaturation episode with oxygen saturation dropping to 60%, accompanied by apnea. Ventilation support was promptly initiated. Over a 30-minute period, the infant had 4 episodes of apnea and desaturation without bradycardia. Consequently, the infant was transferred to the neonatal intensive care unit (NICU) for close monitoring.

**Fig. 1:**
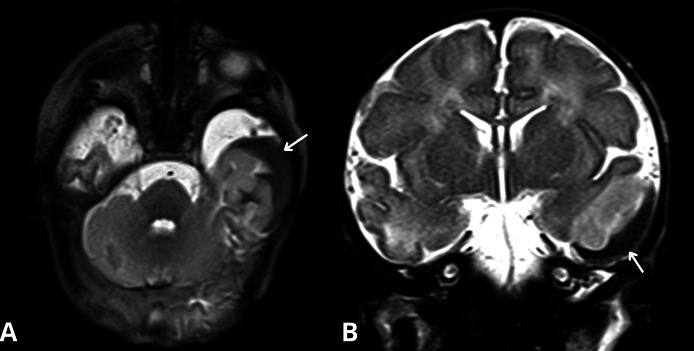
Acute subpial hemorrhage collection in the left temporal region (arrows), shown as T2 hypointensity in axial (A) and coronal (B) views.

**Fig 2:**
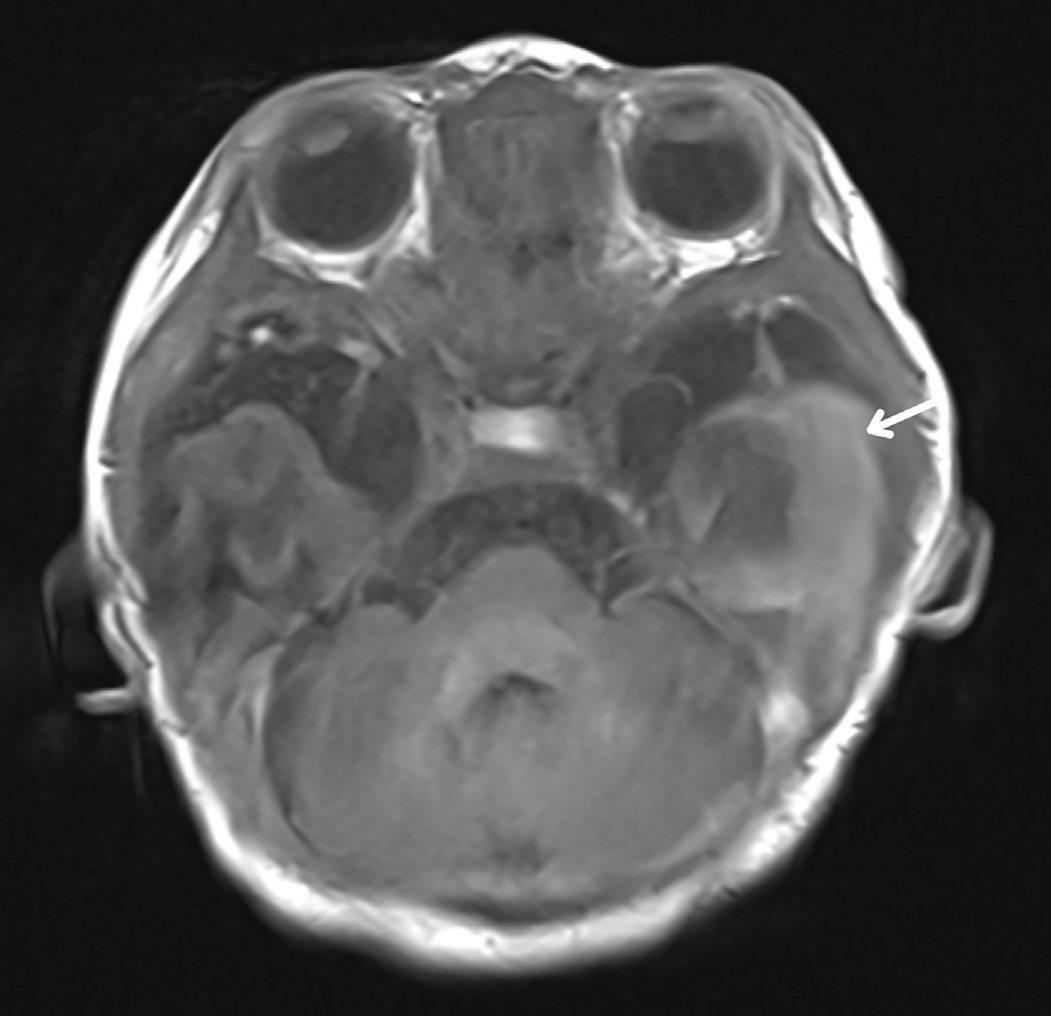
Acute subpial hemorrhage collection in the left temporal region (arrows), shown as T1 hyperintensity in axial views.

**Fig 3:**
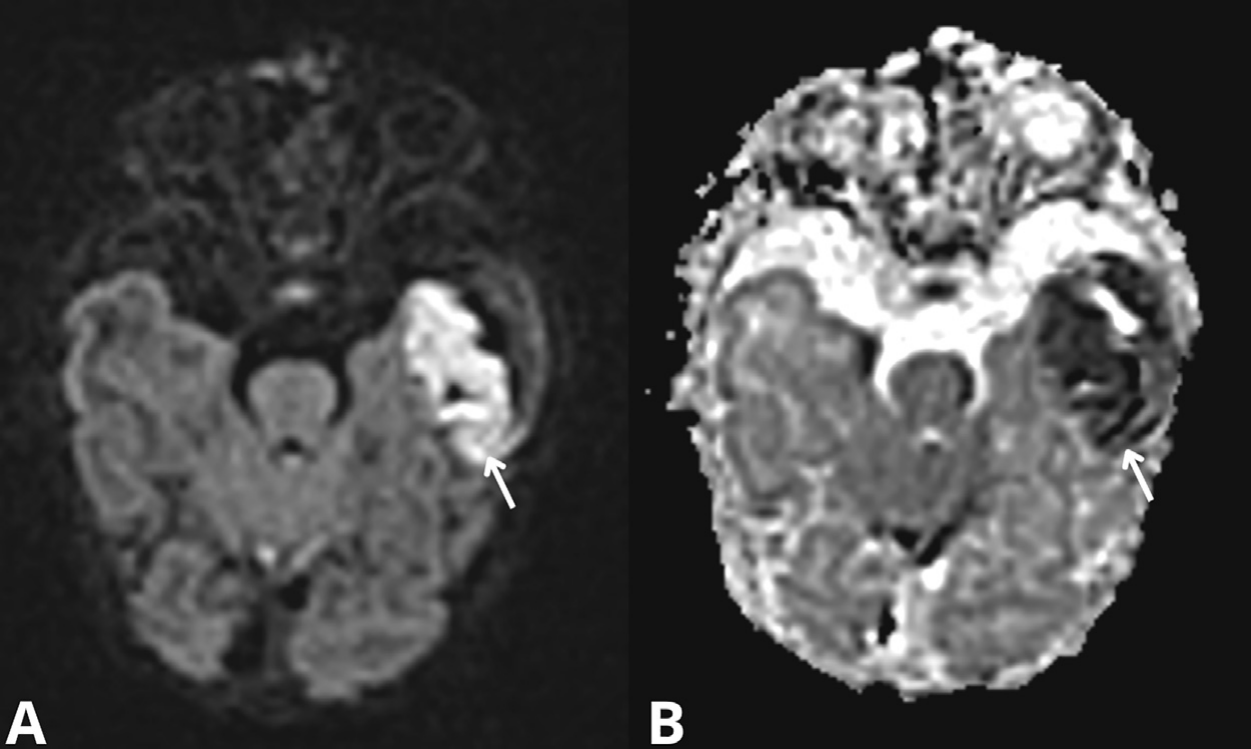
Adjacent cortical cytotoxic edema (arrows), shown as hyperintensity on diffusion-weighted imaging (A) with restricted ADC signal (B).

In the NICU, the infant exhibited signs of seizure activity. The first electroencephalography (EEG) showed subnormal findings. This was followed by a normal transfontanellar ultrasound (ETF). However, an MRI performed, revealed an acute hemorrhagic collection in T1 hyperintensity and T2 hypointensity localized (Fig. 1, Fig 2) in the left temporal region, separated from the subarachnoid spaces on its external border and molding the external cortical surface on its internal border. Adjacent to this, a cortical cytotoxic edema extended into the subcortical white matter in restricted diffusion (Fig. 3).

The infant remained hospitalized for approximately 20 days and was discharged at 41 weeks and 6 days of gestational age, following stabilization and the absence of further seizure activity.

## Discussion

The case presented involves a rare pathology: subpial hemorrhage (SPH). The pia mater is traversed by perforating vessels, making this region particularly vulnerable during direct trauma. Due to its relatively loose structure, the cortex can easily distend in the presence of edema, leading to the rupture of perforating vessels and subsequent hemorrhage.

Clinically, newborns with SPH present with symptoms such as seizures, hypotonia, or severe respiratory distress, as seen in this case. Seizures are the primary presenting symptom, often prompting further neurological evaluation, including EEG and MRI. In our case, the MRI showed an acute hemorrhagic collection in the left temporal region, with characteristic T1 hyperintensity and T2 hypointensity. The adjacent cortical cytotoxic edema observed in this patient is a consistent finding in SPH cases.

SPH is diagnosed based on the presence of a specific radiographic pattern, including a hemorrhagic collection located in the subpial space. This space, situated between the subarachnoid space and the cortical surface, is often overlooked. Interestingly, even small-volume subpial hematomas can be associated with pronounced cortical cytotoxic edema, suggesting a common pathological mechanism rather than a mere mass effect.

In this case, early diagnosis through advanced neuroimaging, including MRI, was crucial for identifying the extent of the hemorrhage and assessing the cortical and subcortical involvement. MRI not only confirmed the presence of subpial hemorrhage but also helped identify the cortical cytotoxic edema and rule out any additional anoxic or ischemic lesions.

While the exact etiology of SPH remains debated, acute fetal distress during labor is considered a significant risk factor. The mechanical forces during delivery may contribute to the rupture of fragile perforating vessels in the superficial cortical layers, leading to hemorrhage. However, in our case, no acute fetal distress was noted during delivery, highlighting that SPH can also occur in the absence of such events.

Fortunately, the clinical and radiological evolution of SPH is generally favorable with appropriate management, as observed in our case. Intensive neonatal care, including anticonvulsant therapy, helped control seizures. Long-term prognosis in similar cases has been variable, with some infants developing epilepsy or other neurological sequelae. However, most cases resolve with minimal long-term impairment, thanks to early intervention and close monitoring.

## Conclusion

This case underscores the importance of recognizing subpial hemorrhage (SPH) as a rare but significant cause of neonatal seizures and respiratory distress. The diagnosis of SPH relies on specific imaging features, such as a well-circumscribed hematoma in the subpial space and cortical cytotoxic edema. Early neuroimaging, particularly MRI, is essential for identifying hemorrhagic and ischemic lesions that might be missed on ultrasound. SPH primarily affects full-term newborns without obvious risk factors, and while the prognosis is generally good with appropriate neonatal intensive care and anticonvulsant therapy, it can be more reserved in cases of large hemorrhages or in preterm infants.

## Patient consent

Written, informed consent was obtained from the parent of the patient for publication of this case.

